# Incidence and management of diaphragmatic palsy in patients after cardiac surgery

**DOI:** 10.4103/0972-5229.43676

**Published:** 2008

**Authors:** Yatin Mehta, Mayank Vats, Ajmer Singh, Naresh Trehan

**Affiliations:** **From:** Department of Anesthesia and Critical Care, Physiotherapy and Cardiac Surgery, Escorts Heart Institute and Research Centre, Okhla Road, New Delhi

**Keywords:** Adult cardiac surgery, chest physiotherapy, diaphragmatic palsy

## Abstract

**Background:**

Diaphragm is the most important part of the respiratory system. Diaphragmatic palsy following cardiac surgery is not uncommon and can cause deterioration of pulmonary functions and attendant pulmonary complications.

**Objectives:**

Aim of this study was to observe the incidence of diaphragmatic palsy after off pump coronary artery bypass grafting (OPCAB) as compared to conventional CABG and to assess the efficacy of chest physiotherapy on diaphragmatic palsy in post cardiac surgical patients.

**Design and Setting:**

An observational prospective interventional study done at a tertiary care cardiac centre.

**Patients:**

2280 consecutive adult patients who underwent cardiac surgery from February 2005 to august 2005.

**Results:**

30 patients out of 2280 (1.31%) developed diaphragmatic palsy. Patients were divided based on the presence or absence of symptoms viz. breathlessness at rest or exertion or with the change of posture along with hypoxemia and / or hypercapnia. Group I included 14 patients who were symptomatic (CABG n=13, post valve surgery n=1), While Group II included 16 asymptomatic patients (CABG n=12, post valve surgery n=4), 9 patients (64%) from Group I (n=14) and 4 patients (25%) from group II showed complete recovery from diaphragmatic palsy as demonstrated ultrasonographically.

**Conclusion:**

The incidence of diaphragmatic palsy was remarkably less in our adult cardiac surgical patients because most of the cardiac surgeries were performed off pump and intensive chest physiotherapy beginning shortly after extubation helped in complete or near complete recovery of diaphragmatic palsy. Chest Physiotherapy led to marked improvement in functional outcome following post cardiac surgery diaphragmatic palsy.

We also conclude that ultrasonography is a simple valuable bed-side tool for rapid diagnosis of diaphragmatic palsy

## Introduction

The respiratory system functions as a vital pump that moves air in and out of the lungs to help in gas exchange. The dome-shaped diaphragm is the most important component of this respiratory muscle pump. It contributes up to 60-70% of the total ventilation at rest both in the sitting and supine positions. The diaphragm is innervated by cervical motor neurons (C3-5) via phrenic nerves.

The incidence and prevalence of diaphragmatic palsy (DP) after cardiac surgery is not exactly defined in the literature, partly because partial or unilateral impairment may go unrecognized. However incidence of diaphragmatic palsy after coronary artery bypass grafting (CABG) ranges from 10% to 60% in English literature. Although in most cases, it is transient and of no clinical significance. But on other hand, diaphragmatic palsy causes further deterioration of pulmonary function in patients with poor cardiopulmonary reserve and may lead to secondary hypoxemia, prolonged ventilator use, pneumonia and atelectasis leading to increased ICU and hospital stay as well as increased morbidity and mortality.

The causes of the diaphragmatic palsy during cardiac surgery are also not clearly understood. Direct injury during harvesting of the internal mammary artery,[1] cold injury owing to pericardial ice slush[2–5] and inadvertent stretch injuries during intra-pericardial manipulation of the heart are some documented causes, but the injury may occur without an apparent reason.

Post cardiac surgery diaphragmatic palsy usually tends to be unilateral but rarely may be bilateral with consequent ventilatory failure. With paralysis of the diaphragm, the patient has to put more effort into breathing, which results in fatigue of the respiratory muscles and may lead to ventilatory failure.

Conventional chest physiotherapy (including coughing, deep breathing exercises and incentive spirometry) may have a beneficial effect in the post operative pulmonary impairment. On the basis of the above hypothesis, an observational prospective interventional study was performed to see the effectiveness of chest physiotherapy in diaphragmatic palsy in post cardiac surgery patients. The other objective of this study was to observe the incidence of diaphragmatic palsy (DP) after off pump CABG (OPCAB) in adult patients.

## Materials and Methods

After approval from the institutional ethics committee and getting informed consents, all consecutive adult patients who underwent cardiac surgery from February 2005 to August 2005, were studied. 30 patients out of 2280 (Off Pump CABG n=1943, cardiac valve surgery n=337) developed diaphragmatic palsy. The diagnosis was suspected on clinico-radiological grounds in patients with respiratory distress and unexplained hypoxemia and /or hypercapnia in the post operative period, with a post operative chest X-ray showing an elevated hemi-diaphragmatic shadow. The diagnosis was subsequently confirmed by ultrasonography. Demonstration of a relatively fixed diaphragm (i.e the diaphragm failed to move or moved paradoxically in a cephalad direction on inspiration. The clinical data and course of the diaphragmatic palsy of these patients were obtained by their hospital records. After confirmation of diaphragmatic palsy, patients were divided in to two groups based on presence or absence of symptoms.

**Group I** - Symptomatic (dyspnoea at rest or minimal exertion, unexplained hypoxemia)

**Group II** - Asymptomatic

Analgesia in both groups was managed by intravenous Tramadol hydrochloride 50-100 mg thrice daily till the maximum dose of 200 mg per day was reached, to remove the bias of different modes of pain relief between the two groups. In all patients, emphasis was given to the optimization of cardiopulmonary status.

### Physiotherapy protocol

All patients were seen by a physiotherapist before surgery, who explained the need to expectorate excess bronchial secretions after surgery. Patients were also taught Huffing, Coughing and deep breathing with sternal support, incentive spirometry and active exercises of the upper and lower limbs.

In addition to this treatment, patients in both groups were treated according to the following protocol in three different phases.

*Phase I (During Intubation):* Chest physiotherapy with endotracheal suction every 2 hourly.

*Phase II (Post extubation):* Local expansion (lateral costal and abdominal), three to four consecutive deep breaths were interspersed between periods of quiet breathing. Patients practiced these exercises in sitting or half lying positions. Humidifier, venturi mask, continuous positive airway pressure / Bi-level positive airway pressure were also used as needed.

*Phase III (Pre discharge):* A fixed set of maneuvers like local expansion, diaphragmatic breathing exercises, deep breathing and Incentive spirometry was taught to the patients and were asked to do the same three to four times a day after discharge, for the next three months. Pulmonary function tests (PFT) were done in both the groups immediately before discharge.

At three months follow-up, a well-designed questionnaire was distributed to all the patients enrolled in the diaphragmatic palsy group to check the frequency of exercises being performed and to assess symptomatic improvement during three month follow up. Chest X-ray interpretation and ultrasonography were done by the same radiologist (to remove the observer bias) to record the magnitude of improvement of diaphragmatic palsy.

## Results

Out of 30 patients, 14 patients (47%)(CABG n=13, post cardiac valve surgery n=1) were symptomatic (Group I) and 16 patients(53%) (12 CABG n=12, post cardiac valve surgery n=4) were asymptomatic (Group II) [Table 1].

**Table 1:** Patients' characteristics *

Variable	Patients with DP (n=30)		
	
	Patients without DP (n=2250)	Symptomatic (n=14) (%)	Asymptomatic (n=16) (%)
Age, (years)	59.3 ± 12.08	57.4 ± 11.06	56.13 ± 10.13
Male: Female ratio	12:3.8	10:4	9:6
LVEF%	43.5 ± 9.83	46.64 ± 8.65	47.53 ± 7.87
Pre-op pulmonary problem	495 (22%)	4 (28.5)	3 (18.75)

* All results are expressed as mean ± SD, LVEF - Left Ventricular Ejection Fraction; n - number of patients, DP-Diaphragmatic palsy

The overall incidence of diaphragmatic palsy was 1.31% among the studied population. 83% (25 patients) had left sided diaphragmatic palsy and 16% (5 patients) had right sided diaphragmatic palsy. Five patients in Group I and one patient in Group II required postural drainage along with chest percussor to encourage expectoration [Table 2].

**Table 2:** Clinical variables *

Variable	Patients without DP (n=30)		
	
	Patients with DP n=2250	Group I (n=14) (%)	Group II (n=16) (%)
Mechanical ventilation (hours) mean ± SD	12.2 ± 7.43	22.79 ± 19.51	20 ± 15.56
Pulmonary complication (n) %	57 (2.53%)	5 (35.7)	1 (6.25)
Chest Infection (n) %	12 (0.5%)	0 (0)	0 (0)
Mortality (n) %	7 (0.31%)	0 (0)	1 (6.25)

* DP-Diaphragmatic palsy

*Pulmonary Complications:* The incidence of pulmonary complication (viz. atelectasis, retained secretions) was 36% (n=5) for group I and 6.25% (n=1) for Group II.

*Oxygenation Parameters:* In phase I, oxygenation parameters were same for both the groups. In phase II, parameters were better for Group II than Group I. In phase III again better parameters were recorded for Group II than Group I, although no statistical significance could be established due to small sample size [Table 3].

**Table 3:** Clinical oxygenation parameters in 3 phases *

Variable	Phase I (intubation)	Phase II (extubation)	Phase III (pre-discharge)
**Group I (n=14)**			
**FiO_2_60**%			
PaO_2_ mm Hg	156 ± 34.09	136.07 ± 48.74	73.93 ± 19.6
PaCO^2^ mm Hg	38.64 ± 4.09	40.14 ± 4.38	36.04 ± 2.92
SaO_2_%	99.5 ± 1.16	98.79 ± 1.97	94.96 ± 3.85
RR	15.21 ± 2.86	20.14 ± 1.61	20.56 ± 2.42
**Group II (n=16)**			
**FiO2 60**%			
PaO_2_ mm Hg	150.75 ± 25.62	176.75 ± 55.79	77.03 ± 11.96
PaCO_2_ mm Hg	38.42 ± 4.48	39.8 ± 2.52	34.19 ± 3.25
SaO_2_%	99.50 ± 0.89	99.63 ± 0.89	96.24 ± 1.95
RR	16.75 ± 2.82	21.31 ± 2.52	19.63 ± 1.44

• All results are expressed as mean ± SD, (PaO_2_) Partial pressure of oxygen in arterial blood, (PaCO_2_) Partial pressure of carbon-di-oxide in arterial blood; (SaO^2^%) saturation % of oxygen, (RR) respiratory rate.

*Pulmonary function test:* Forced vital capacity (FVC), forced expiratory volume in 1 sec.(FEV_1)_, FEV_1_ /FVC, peak expiratory flow rate (PEFR), peak inspiratory flow rate (PIFR) were compared and found to be better for Group II [Table 4].

**Table 4:** Pulmonary function test value comparison *

Variable	Patients without DP n=2250	Patients with DP (n=30) Group I (n=14)	Group II (n=16)
FVC (% Pred)	62.3 ± 16.28	39 ± 10.5	44.29 ± 8.18
FEV1 (%Pred)	55.7 ± 11.16	44.57 ± 10.9	47.14 ± 9.08
FEV1 /FVC	89.40 ±15.8	121.14±18.9	109.57 ± 5.5
PEFR (% Pred)	68.2 ± 17.1	49.29 ± 21.3	61.8 ± 25.94
PIFR (% Pred)	65.61 ±13.32	47.43 ± 27	52.8 ± 24.5

• DP-Diaphragmatic palsy, • All results are expressed as mean ±SD, • FVC - Forced vital capacity (% Pred), FEV1- forced expiratory volume in 1 sec (% Pred), PEFR - peak expiratory flow rate (% Pred), PIFR - peak inspiratory flow rate (% Pred)

Table 5 reveals comparative analysis of PFT at three time intervals (Preoperative, pre discharge and three months follow-up)

**Table 5:** Pulmonary function test variables in both groups at different time interval

PFT variable	Group I (n=14)	Group II (n=16)				
		
	Preoperative	At discharge	3 month follow up	Preoperative	At discharge	3 month follow up
FVC % pred.	63.2±12.1	39±10.5	54.7±8.68	61.8±9.0	44.2±8.18	57.3±9.16
FEV1 % pred.	58.1±8.69	44.5±10.9	51.8±12.48	56±13.2	47.14±9.08	54.1±8.76
FEV1/FVC	91.93±12.8	121.14±18.9	94.69±11.18	90.61±17.6	109±5.5	94.41±9.21
PEFR % pred	64.1±10.2	49.29±21.3	57.3±18.23	63.4±18.6	61.8±25.9	62.6±21.4
PIFR %pred	61±8.9	47.43±27	54±11.6	64.2±16.3	52.8±24.5	61.3±18.5

*Follow-up (After three months):* 85% pts (n=12) from Group I and 75% patients (n=12) from Group II performed the exercise regime at home regularly as advised. 64% patients (n=9) from Group I and 25% patients (n=4) from Group II showed complete recovery from diaphragmatic palsy as demonstrated by ultrasonographically. This reveals objective improvement and documents the efficacy of postoperative chest physiotherapy in cases of diaphragmatic palsy.

All patients from Group I showed symptomatic improvement and led a normal healthy lifestyle.

## Discussion

In normal adults, diaphragm excursion may contribute 30% to 60% of the total minute ventilation.[6] With unilateral diaphragm palsy, there is decrease of 20% to 30% of vital capacity and maximum voluntary ventilation and a 20% decrease in oxygen uptake on the affected side.[7] Adult patients with diaphragm palsy can generally be weaned from mechanical ventilation because of the compensation from the intercostal muscles or the accessory muscles of respiration.

The main cause of diaphragm palsy after adult cardiac surgery is attributed to the topical cooling.[2,3,4] Other causes include cutting, detrition, traction, and thermal burns from the electric knife.[8] Some reports have explained the relationship of the use of internal mammary artery (IMA) and postoperative phrenic nerve dysfunction in patients with CABG.[9] The phrenic nerves cross the internal mammary artery anteriorly in 54% of cases and posteriorly in 14%.[10] Phrenic nerve injury can also result from injury to the pericardiophrenic artery, in addition to the direct injury. Phrenic nerve regeneration is estimated at a rate of 1 mm/day.[11] Cohen *et al*, noted that phrenic nerve recovery occurs at least partially in 75% to 90% of the patients[8] which is similar to our findings. Kuniyoshi *et al*, observed many patients of diaphragmatic palsy who were absolutely asymptomatic and could be weaned off from the ventilator easily. They considered diaphragmatic palsy as a significant cause of postoperative respiratory dysfunction.

Diaphragmatic palsy is usually suspected when diaphragmatic elevation is seen on the X-ray chest. However, in the postoperative period, with the patient in the supine position and on ventilatory support, it may not be easily detected because positive pressure ventilation tends to mark abnormal findings. Furthermore, in the spontaneously breathing patients, common sequel of cardiac surgery e.g. left pleural effusion, lower lobe atelectasis and elevation of the hemidiaphragm can mask phrenic nerve injury. Confirmatory tests for the diagnosis of diaphragmatic palsy include esophageal and gastric pressure measurements, fluoroscopy, ultrasonography, and electro-neuromyography.[9] The diagnosis of the diaphragmatic palsy should be made on the basis of the results of the multiple examinations including the symptoms.

The incidence of pulmonary complications was although numerically higher in patients in group I and group II but it was not significantly different from patients without diaphragmatic palsy owing to early recognition of diaphragmatic palsy and preventive measures taken to avoid respiratory complications (i.e. chest physiotherapy, steam inhalation, CPAP, BiPAP as required.) The incidence of postoperative respiratory infections e.g. tracheal bronchitis, Ventilator associated pneumonia, hospital acquired pneumonia etc were significantly low compared to historical controls in our observation group as well as study group. This may be clearly attributable to all surgeries being performed Off Pump CABG hence facilitating fast track extubation and expertise of our dedicated team in postoperative cardiac critical care owing to large number of surgeries done per year. Also, early anticipation and all necessary actions to prevent the development of postoperative pulmonary infection contributed to reduced incidence of above complications.

Objective improvement in PFT parameters when compared at three time intervals revealed that there was remarkable decline in PFT values in patients with diaphragmatic palsy as compared to preoperative values, but after vigorous chest physiotherapy the PFT values return back to near preoperative values, indicating significant improvement in lung function in both the groups. We did not collect data at the time of discharge and follow-up of patients without diaphragmatic palsy because it was a large group and due to cost and resource considerations.

At three months, our study showed improvement in lung function in both the groups, which was attributed to regular physiotherapy exercises. For ethical reasons, it was not possible to include a control group of patients with diaphragmatic palsy who received no physiotherapy treatment. Conversely it was also not considered worthwhile to compare the effects of chest physiotherapy in patients with or without diaphragmatic palsy.

Most patients with post-cardiac surgery diaphragmatic dysfunction improves with conservative measure such as chest physiotherapy, prevention and treatment of pneumonia, optimum treatment of underlying pulmonary disease and optimization of cardiovascular / hemodynamic and oxygenation parameters. Same was true for marked and sustained improvement of patients in our study.

## Conclusion

There was subjective as well as objective improvement in functional outcome following diaphragmatic palsy in both the groups. Chest physiotherapy helps to preserve lung function and hasten recovery from diaphragmatic palsy. Our study also emphasizes that ultrasonography is a simple and effective bed side tool in ICU to rapidly diagnose diaphragmatic palsy and hence avoiding cumbersome methods e.g. trans diaphragmatic pressure measurement or fluoroscopy which are also less available in many hospitals. Early diagnosis and prevention of pulmonary complications along with intensive physiotherapy leads to good outcome in patients with diaphragmatic palsy.

Based on our observation, a well designed randomized controlled trial is recommended in order to establish the role of chest physiotherapy after post cardiac surgery diaphragmatic palsy.
